# Response of *Arabidopsis thaliana* Roots with Altered Lipid Transfer Protein (LTP) Gene Expression to the Clubroot Disease and Salt Stress

**DOI:** 10.3390/plants5010002

**Published:** 2015-12-24

**Authors:** Sabine Jülke, Jutta Ludwig-Müller

**Affiliations:** Institut für Botanik, Technische Universität Dresden, Dresden 01062, Germany; Jutta.Ludwig-Mueller@tu-dresden.de

**Keywords:** *Arabidopsis thaliana*, clubroot disease, lipid transfer protein, salt stress, osmotic stress, *Plasmodiophora brassicae*, root growth

## Abstract

The clubroot disease of Brassicaceae is caused by the obligate biotrophic protist *Plasmodiophora brassicae*. The disease is characterized by abnormal tumorous swellings of infected roots that result in reduced drought resistance and insufficient distribution of nutrients, leading to reduced crop yield. It is one of the most damaging diseases among cruciferous crops worldwide. The acquisition of nutrients by the protist is not well understood. Gene expression profiles in *Arabidopsis thaliana* clubroots indicate that lipid transfer proteins (LTPs) could be involved in disease development or at least in adaptation to the disease symptoms. Therefore, the aim of the study was to examine the role of some, of the still enigmatic LTPs during clubroot development. For a functional approach, we have generated transgenic plants that overexpress *LTP* genes in a root specific manner or show reduced *LTP* gene expression. Our results showed that overexpression of some of the *LTP* genes resulted in reduced disease severity whereas the lipid content in clubs of LTP mutants seems to be unaffected. Additional studies indicate a role for some LTPs during salt stress conditions in roots of *A. thaliana*.

## 1. Introduction

Plant lipid transfer proteins (LTPs) were defined by their ability to transfer phospholipids between membranes *in vitro* [1]. Since their discovery 40 years ago [2], many investigations were performed to understand their structure and biological function, but up to now their role in plants is still ambiguous.

Plant LTPs are small (6.5–10.5 kDa) basic proteins with an isoelectric point between 8.5 and 12 [3]. A crucial feature is the so-called eight cysteine motif that comprises eight cysteine residues located at conserved positions, which can form four disulfide bridges [1,3]. The motif results in a tertiary structure of conserved alpha helices that are connected with variable loops, thereby forming the four disulfide bridges in a specific manner [4].

LTPs are encoded by large multigene families [3,5], but only for a few of the encoded proteins was the lipid transfer activity demonstrated. The *LTP* gene family *in Oryza sativa* contains 52 members [3], in *Triticum aestivum* 156 members [3] and in *Arabidopsis thaliana* 110 members, of which 15 are named *LTP1* to *LTP15* [5] and the others are grouped as lipid transfer proteins (TAIR database [6]). Based on the complexity of the gene families, a systematic classification is difficult and there is not a single one commonly used and accepted. Groupings were done based on the *in vitro* activity of the corresponding enzymes, mainly lipid binding [7], based on sequence homologies [3], depending on biochemical and structural properties [8,9], or based on phylogenetic aspects [3].

Up to now, many possible functions for LTP proteins were discussed including a role during adaptation to biotic and abiotic stress factors. There are many indications that LTPs play a role in plant defense. Firstly, many examples show that *LTP* gene expression is induced in response to pathogen infection, but there are also cases where *LTP* gene expression is repressed [10,11,12]. Secondly, there are some examples indicating that overexpression of a *LTP* gene caused resistance to pathogen infection [13,14].

The mode of action of LTPs in the induction of disease resistance is not fully understood. It was shown that some LTPs have an antimicrobial activity and can directly act against the pathogen [12,15]. Moreover, some LTPs lead to permeabilization of the pathogen membrane [16,17], but this latter feature does not correlate with the ability to bind lipids [16]. In addition, it was shown that some LTPs can bind to an elicitin receptor [18], which led to the hypothesis that they are involved in signaling processes after pathogen attack. Furthermore, some LTPs can bind calmodulin [19,20], which is a prominent calcium sensor, and others can be phosphorylated [21,22], both being common features of plant signaling pathways.

Among abiotic stress factors, salt stress affects plant growth and development in various ways, so soil salinity is one of the major obstacles in the environment [23]. High salt stress is also the cause of osmotic stress. Also, similarities between drought and salt stress have been summarized [24] and the response pathways in many cases share similar signaling components [25]. To reduce the inhibitory effects of salinity on plant growth, the question which factors are involved in salt tolerance play a major role. Many factors have been identified including ion homeostasis, osmolytes, signaling components, transcription factors and protective proteins [23]. Based on gene expression data, there is also plenty of evidence that LTPs are somehow involved in adaptation to salt stress [26,27,28], osmotic stress [27,29], drought stress [10,26,30] and cold stress [10,31].

The clubroot disease of Brassicaceae is caused by the obligate biotrophic protist *Plasmodiophora brassicae*. It is worldwide one of the most damaging diseases within this plant family. The model plant *A. thaliana* is a good host for *P. brassicae* infection, because it develops all typical clubroot symptoms. The resting spores of *P. brassicae*, which have a half-life of 3.6 years [32], survive up to 15 years in the soil [33]. During the primary phase of the life cycle, the pathogen infects root hairs and develops into secondary zoospores [34], which subsequently infect the root cortex (secondary phase). While almost no disease symptoms are visible during the primary infection, the main changes in root morphology occur during the secondary infection. Thereby, the characteristic galls develop as a result of continuous cell division and enlargement. Infected cells are completely filled with the resting spores of the pathogen and can be up to 10 times larger than non-infected cells [35]. During this late phase of infection, the characteristic root architecture is completely destroyed leading to disordered nutrient and water uptake and distribution. In addition, the changes in root morphology are accompanied by a plethora of physiological changes. Auxins and cytokinins are involved in the regulation of cell division and enlargement [36]. Moreover, cytokinins are responsible to establish a metabolic sink to ensure the nutrition of the pathogen [37]. Mainly trehalose [38] and soluble sugars as well as starch [38] accumulate in galls and other infected tissues of *A. thaliana*. Scanning electron microscopy reveals starch and lipids as storage compounds in almost all developmental stages of the pathogen [39].

There are only a few data about the general changes on RNA and proteins in response to clubroot infection. Proteome analyses were done with *Brassica napus* roots 24 and 72 h after inoculation [40] and using *A. thaliana* roots at four days after inoculation [41]. Changes were observed in proteins involved in metabolism, cell defense, calcium homeostasis and detoxification of reactive oxygen species [40,41]. Transcriptome analyses were done on infected *A. thaliana* roots 4, 7 and 10 days after inoculation [42] and 10 and 23 days after inoculation [11]. On the one hand, it was shown that four days after inoculation the expression of genes that participate in pathogen perception and signaling in resistant host–pathogen interactions was induced [42]. On the other hand, the expression of genes involved in defense reactions was repressed. This was also the case for many members of the LTP family [11].

Based on the observation that *LTP* genes with their many possible functions, especially in pathogen defense, show (a) differential expression during clubroot; and (b) overexpression of *LTP* genes in other work results in resistance to other plant pathogens, we speculated that LTPs could also be involved in clubroot disease development. Therefore, the aim of this study was to investigate the role of selected LTPs from *A. thaliana* during clubroot development. Based on microarray expression data, nine *LTP* genes (*LTP1*, *LTP3*, *LTP4*, *AT1G12090*, *AT1G62510*, *LTP8*, *AT3G22620*, *AT4G33550*, *AT5G05960*) were selected for further investigation. First, their expression levels were monitored and second, functional analyses were performed. Here, mutants and overexpressor lines for the corresponding genes were analyzed with regard to various root phenotypes including clubroot infection, lipid composition, as well as salt and osmotic stress conditions.

## 2. Results and Discussion

### 2.1. LTP Gene Expression Is Differentially Regulated during Clubroot Infection

**Figure 1:**
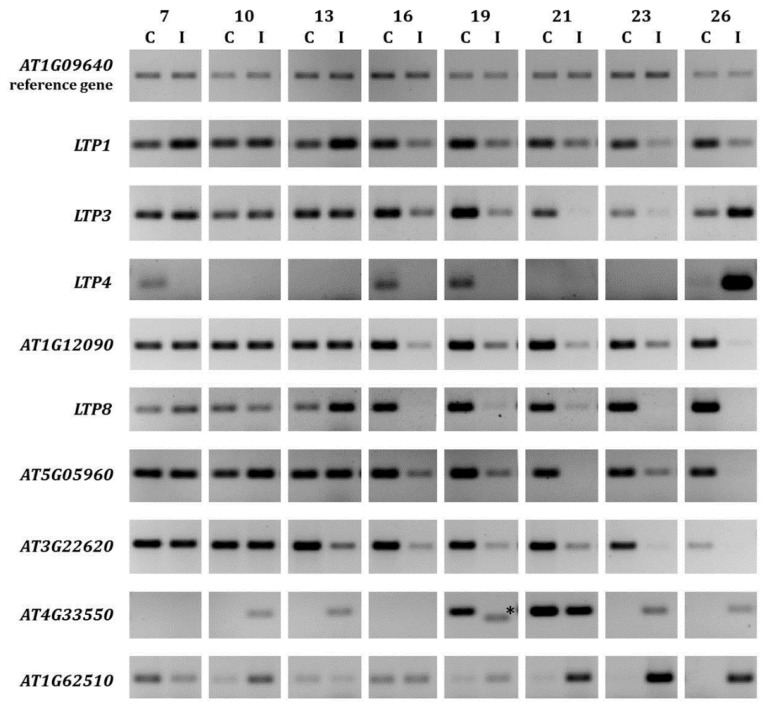
Expression of selected *LTP* genes in clubroot infected *A. thaliana* roots. The expression for selected *LTP* genes during clubroot infection between seven and 26 days after inoculation is shown compared to control roots of the same age. For infection, the single spore isolate e_3_ was used. At least two technical replicates were done to confirm the results. For each analyzed time point, approximately 30–50 plants were used for RNA extraction. The asterisk marks an unspecific PCR product. C = control (not inoculated); I = inoculated (infected).

Many reports describe the differential regulation of *LTP* gene expression due to infection with various pathogens, including fungi and bacteria [10,12]. Re-evaluation of previous microarray data from clubroot infected roots [11] showed that *LTP* genes were strongly differentially regulated at two time points during infection (Table 1). Therefore, we have analyzed the *LTP* gene expression from the nine *LTP* genes showing the strongest regulation (Table 1) throughout a more detailed time frame between seven and 26 days after inoculation (Figure 1).

**Table 1:** Overview of *LTP* genes analyzed in this study.

10 dai	23 dai	Locus	Description	Name	LTP Mutants Used	Fold-Regulation of Transcript Levels for Altered *LTP* Gene
**−50.0**	**445.5**	AT2G38540	Non-specific lipid transfer protein	*LTP1*	*LTP1-OX*	2.6 ↑
**−1.2**	**87.1**	AT5G59320	Predicted to encode a PR (pathogenesis-related) protein. Belongs to the lipid transfer protein (PR-14) family	*LTP3*	*LTP3-OX* *LTP3-KO*	4.2 ↑ 20.3 ↓
**1.2**	**539.2**	AT5G59310	Encodes a member of the lipid transfer protein family	*LTP4*	*LTP4-OX* *LTP4-KO*	10.5 ↑ 3.5 ↓
**−4.8**	**−14.3**	AT1G12090	Extensin-like protein		*AT1G12090-OX * *AT1G12090-KO*	1.5 ↑ 2.7 ↓
**−2.7**	**−10.0**	AT2G18370	Predicted to encode a PR (pathogenesis-related) protein. Belongs to the lipid transfer protein (PR-14) family	*LTP8*	*LTP8-OX* *LTP8-KO*	4.6 ↑ 8.6 ↓
**1.2**	**−25.0**	AT5G05960	Bifunctional inhibitor/lipid-transfer protein/seed storage 2S albumin superfamily protein		*AT5G05960-KO*	20.9 ↓
**−3.2**	**−14.3**	AT3G22620	Bifunctional inhibitor/lipid-transfer protein/seed storage 2S albumin superfamily protein		*AT3G22620-KO*	1.6 ↓
**4.5**	**4.6**	AT4G33550	Bifunctional inhibitor/lipid-transfer protein/seed storage 2S albumin superfamily protein		*AT4G33550-AS*	10.3 ↓
**−1.5**	**103.1**	AT1G62510	Bifunctional inhibitor/lipid-transfer protein/seed storage 2S albumin superfamily protein		*AT1G62510-AS*	4.7 ↓

The Table shows the regulation of *LTP* gene expression in response to *P. brassicae* infection 10 days after inoculation (10 dai) and 23 days after inoculation (23 dai). Data from a microarray [11] were re-evaluated for LTP genes. Additionally the LTP mutant lines (M) used in this study are listed, whereas OX indicates overexpression of the gene; KO indicates the gene disruption due to T-DNA insertion leading to reduced gene expression (knockdown) or complete repression of gene expression (knockout) and AS indicate gene silencing via antisense RNA. The transcript analyses for the mutant lines were done using semi-quantitative RT-PCR and the program Image J to calculate the regulation of transcript levels by determining the intensity of the respective PCR product.

At seven days after inoculation, only minor changes in cell and root morphology occurred and small plasmodia were the dominant stage of the pathogen [11]. At 14 days after inoculation, the secondary plasmodia were growing and the gall development started. Consequently, the infected cells became hypertrophied and resting spore development was observed. Swollen infected root cells that contained a lot of resting spores dominated the root tissue 24 days after inoculation and large galls were clearly visible.

The results of our expression analyses revealed that from 16 days after inoculation, most of the *LTP* genes showed a reduced expression in clubroot infected roots (Figure 1). Especially, *AT1G12090*, *AT5G05960* and *AT3G22620* gene expressions were exclusively repressed. The expressions of the other *LTP* genes analyzed showed a more inconsistent regulation pattern. For example, *LTP1* gene expression was induced between seven and 13 days after inoculation, but repressed from 16 days after inoculation. *AT4G33550* and *AT1G62510* seemed the only genes where the upregulation during infection dominated. In addition to these data from plants that were infected with the single spore *P. brassicae* isolate e_3_ [43], the *LTP* regulation in plants infected with a field isolate [44] showed the same trend (Supplementary Figure S1). Moreover, it was shown that the differential gene regulation occurred almost throughout the whole infection cycle. While most of these results are in accordance with the microarray data [11], some of the regulation patterns (Figure 1) show differences to the transcriptome data (Table 1). However, it is known that variations in transcription levels between microarrays and subsequent PCR on the same samples can occur [45].

LTPs belong to the so-called pathogenesis-related proteins (PR14). Typically, PR gene expression is induced upon pathogen infection [46]. Therefore, the down-regulation of the *LTP* genes in response to clubroot infection is not as expected for PR genes, but is a common feature for gene regulation patterns altered by obligate biotrophic pathogens [47]. Moreover, there is not much information if and how the plant defends against the *P. brassicae* infection. Only a few proteins and genes that are involved in pathogen perception and cellular defense were found to be induced upon clubroot infection [11,41,42].

It can be speculated that those *LTP* genes, whose expression is repressed, are somehow involved in pathogen defense. Those proteins with induced gene expression like *AT4G33550* and *AT1G62510* are perhaps involved in lipid transfer to the pathogen, because lipid droplets accumulate in various pathogen stages [39,48] and might play a role as a lipid source for *P. brassicae*.

**Figure 2:**
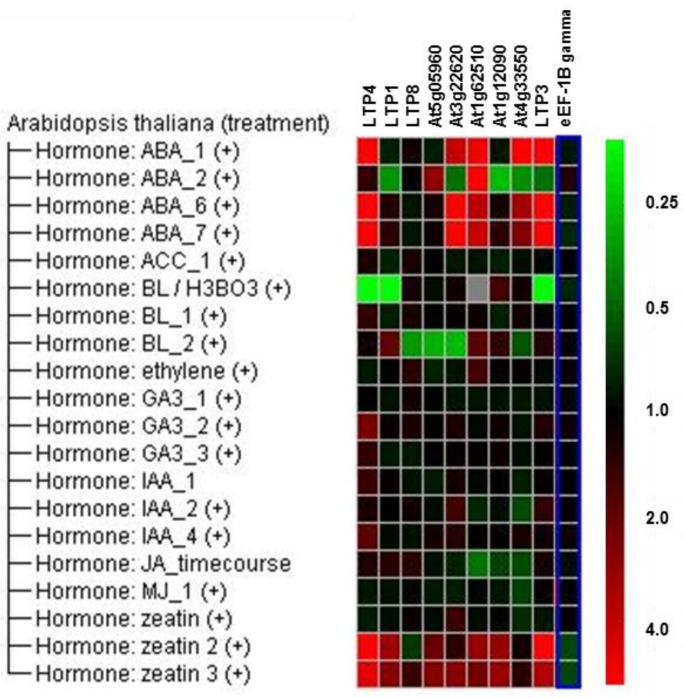
Regulation of *LTP* gene expression due to hormonal treatment according to Genevestigator [49] data. The elongation factor 1B gamma is shown as a reference gene in comparison to the analyzed *LTP* genes. Green color indicates repression and red induction of gene expression.

Genevestigator [49] data (Figure 2) reveal that some *LTP* genes, for example *LTP3* and *LTP4*, are induced by abscisic acid (ABA) and zeatin, whereas brassinolide seems to repress the expression of *LTP1*, *LTP3* and *LTP4*. Auxin, ethylene, gibberellin and jasmonate do not regulate the expression of the *LTP* genes, which were chosen for our study, significantly. Linking these hormones to gene expression during the clubroot disease (Figure 1) showed that the direction of gene regulation did not match the changed hormone level in clubroots. For auxins, cytokinins and ABA, it is known that they increase during clubroot development [37]. Zeatin and ABA induce *LTP* gene expression, but clubroots show a reduced *LTP* gene expression. This indicates that these plant hormones are most likely not the factor responsible for the *LTP* gene regulation during clubroot. Only the patterns for the regulation by brassinosteroids and clubroot seem similar, indicating a possible connection.

Nevertheless, the observation that ABA, a well-known player during various abiotic stress responses [50], can induce the expression of some *LTP* genes seems interesting, since late stages of the clubroot disease are accompanied by drought stress-like symptoms. Therefore, the response of LTP mutants to salt and osmotic stress was also investigated (see Section 2.4).

### 2.2. Overexpression of Some LTP Genes Cause Altered Disease Development Whereas Knockout Has No Effect on Clubroot Development

Based on their strong differential expression during clubroot development, we hypothesized that the proteins encoded by these *LTP* genes can be involved in disease development. Therefore, they are promising candidates to manipulate the outcome of the disease. We have selected a set of *LTP* genes for further functional investigations (Table 1). Based on these data, we have generated transgenic *A. thaliana* plants that overexpress or silence the naturally occurring regulation during pathogen infection. To rule out that an overexpression in the whole plant could cause secondary effects, we have used the root specific promoter pyk10 [51] in our constructs. In addition, we have also analyzed T-DNA insertion lines for several *LTP* genes (Table 1; see Supplementary Table S2 for the site of T-DNA insertion). All plants from the different *LTP* gene mutants and transgenic lines did not show any obvious phenotypical changes compared to the wild type when grown under control conditions (data not shown).

To verify the overexpression (OX) or the reduced expression (KO in the name of the respective lines stands for either knockout or knockdown; AS stands for antisense constructs used) of the respective *LTP* gene we have performed semi-quantitative RT-PCR from 24-day-old *A. thaliana* roots (Table 1, Supplementary Figure S2). For each *LTP* gene, more than one overexpressor line was generated and analyzed with regard to their gene expression level. For further analysis, the line with the strongest *LTP* overexpression was selected.

The transgenic LTP lines *LTP1-OX*, *LTP3-OX*, *LTP4-OX*, *AT1G12090-OX* and *LTP8-OX* showed a higher mRNA amount of the respective *LTP* gene in comparison to the wild type roots (Table 1, Supplementary Figure S2). In the *LTP* T-DNA insertion lines *LTP8-KO*, *AT5G05960-KO* and *LTP3-KO,* the corresponding *LTP* mRNA was not detectable, whereas in *AT1G12090-KO*, *AT3G22620-KO* and *LTP4-KO,* the corresponding *LTP* mRNA was still present, but at a lower level compared to the wild type. Due to the low expression levels of *AT4G33550* and *AT1G62510* in *A. thaliana* roots, we have analyzed the gene expression in young leaves to verify the *LTP* gene down-regulation in the antisense lines *AT4G33550-AS* and *AT1G62510-AS* (Table 1).

Since *LTP* genes form a multigene family, it can be assumed that the altered expression of one *LTP* gene caused changes in the expression of other family genes. Therefore, we have also examined the expression of other *LTP* genes in the mutants. The results showed that the modulation of one *LTP* gene indeed caused changes in the expression of other *LTP* genes (Supplementary Figure S2). Such compensation within a gene family is reasonable and should be taken into account, but we could not find a correlation between a specific mutation and a change in the expression pattern of other *LTP* genes. Interestingly, we found that in the *LTP3*-knockout (*LTP3-KO*) plants *LTP4* expression was induced, whereas *LTP4* was repressed in plants that overexpress *LTP3* (*LTP3-OX*, Supplementary Figure S2). This observation, together with the assumption that *LTP3* and *LTP4* are the result of gene duplication [5], led to the speculation that these proteins have similar functions in plants.

**Figure 3:**
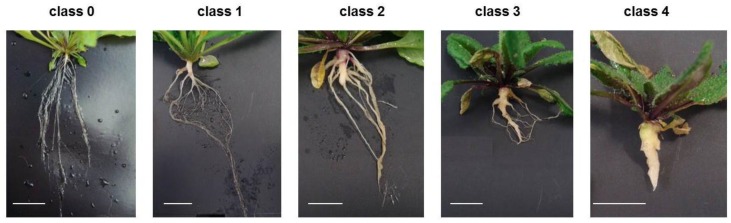
Typical disease symptoms from club-rooted *A. thaliana* for the different disease classes. Disease classes range from 0 = no symptoms visible to 4 = no fine root system present, but one large root gall. For detailed description of symptoms, see Section 3.2. Bar = 1 cm.

The main question of our work was whether the modulation of *LTP* gene expression could result in altered disease development at the best clubroot tolerant plants. To test this hypothesis, infection tests were carried out using *LTP* mutants and overexpressor lines. For a functional analysis, *A. thaliana* plants were inoculated with resting spores from *P. brassicae* and the disease symptoms were rated 26–28 days after inoculation. The rating was based on assigning the infected plants into different disease classes based on their symptom development. The calculated disease index can have a maximum score of 100 (see Section 3.2), which means that all plants show the strongest disease symptoms (class 4, Figure 3). Thus, a high disease index shows susceptibility, whereas a low disease index indicates tolerance. In addition, we calculated the shoot index, which is the ratio between shoot fresh weights of infected plants to the shoot fresh weight of non-infected plants. This shoot index can also be used to evaluate susceptibility or tolerance. A high shoot index reflects a higher vitality of the infected plants, whereas a low shoot index is indicative of more susceptible plants.

**Figure 4:**
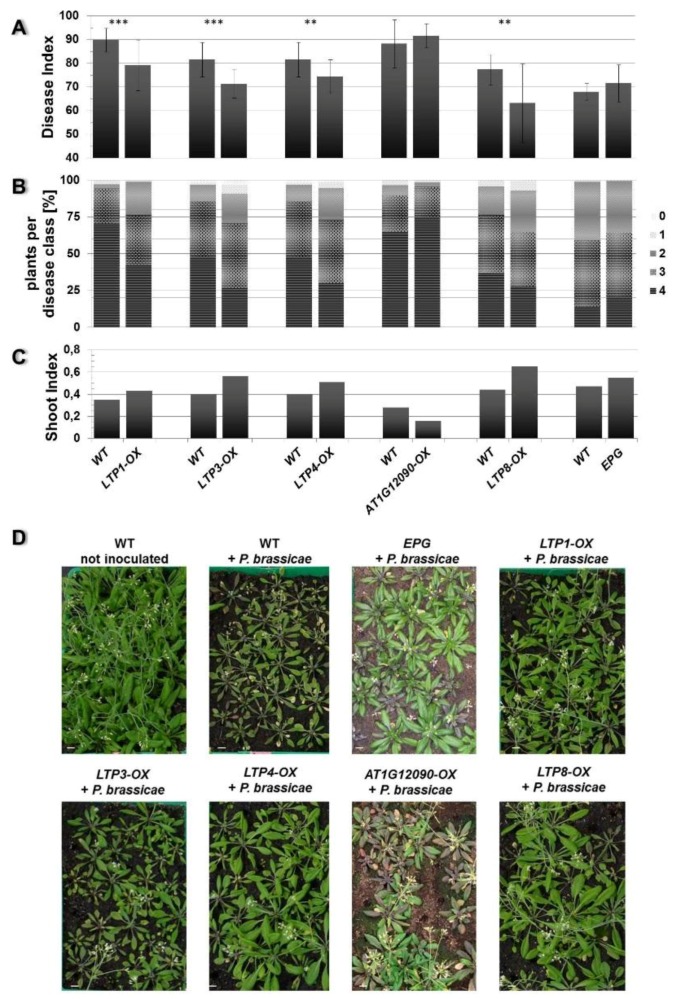
Clubroot development in plants that overexpress a *LTP* gene. The Disease Index (**A**), distribution of disease classes (**B**), and Shoot Index (**C**) for mutants that overexpress (OX) the indicated *LTP* gene in comparison to wild type (WT) are shown. Each mutant line was tested in single experiments with wild type as comparison. Therefore, each mutant line is shown in comparison with its own wild type graph to evaluate the disease development. Each disease index bar value shows the mean value ± SE. The Shoot Index also represents the mean value, but because all analyzed plants were pooled for measuring fresh weight and the calculation of the shoot index, the calculation of the SE was not possible. Mean values were calculated from at least two independent experiments with approximately 100 plants per line. Asterisks indicate a significant difference (for ** *p* < 0.01; *** *p* < 0.001). The growth comparison of the aboveground parts of *P. brassicae* infected *A. thaliana* plants 26 days after inoculation and control (not inoculated) plants is shown in (**D**). The pictures show plants from one representative experiment for all tested mutant lines and wild type. OX: overexpression of the indicated *LTP* gene (*LTP1*, *LTP3*, *LTP4*, *AT1G12090*, *LTP8*), WT: wild type, EPG: empty vector control, bar = 1 cm.

**Figure 5:**
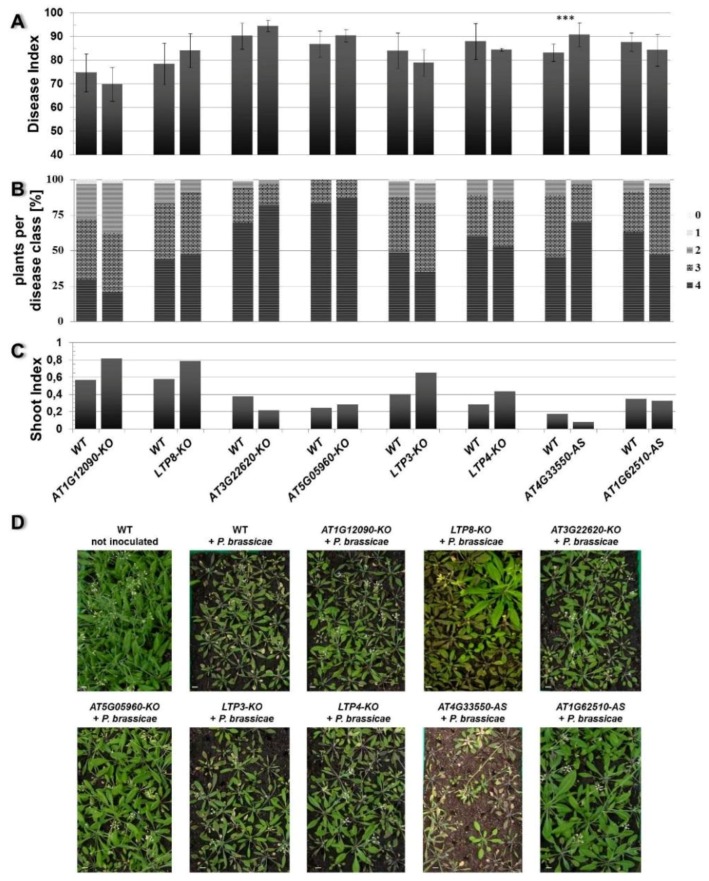
Clubroot development in plants with reduced *LTP* gene expression. The Disease Index (**A**), distribution of disease classes (**B**), and shoot index (**C**), for T-DNA insertion mutants (KO) and antisense lines (AS) for the indicated *LTP* gene in comparison to wild type plants (WT) are shown. Each mutant line was tested in single experiments with wild type as comparison. Therefore, each mutant line is shown in comparison with its own wild type graph to evaluate the disease development. Each disease index bar value shows the mean value ± SE. The Shoot Index also represents the mean value but because all analyzed plants were pooled for measuring fresh weight and the calculation of the shoot index, the calculation of the SE was not possible. Mean values were calculated from at least two independent experiments with approximately 100 plants per line. Asterisks indicate a significant difference (for *** *p* < 0.001). The growth comparison of aboveground parts of *P. brassicae* infected *A. thaliana* plants at 26 days after inoculation and not inoculated plants is shown in (**D**). The pictures show plants from one representative experiment for all tested mutant lines and wild type. KO: T-DNA insertion in the indicated *LTP* gene (*AT1G12090*, *LTP8*, *AT3G22620*, *AT5G05960*, *LTP3*, *LTP4*), AS: silencing of the indicated gene (*AT4G33550*, *AT1G62510*) using the antisense technique, WT: wild type, bar = 1 cm.

Based on these criteria we observed an altered disease development in the LTP overexpressors *LTP1-OX*, *LTP3-OX*, *LTP4-OX* and *LTP8-OX*. These plants showed a decreased disease index and concomitantly a slightly higher shoot index compared to wild type plants (Figure 4). This reduced disease index was the result of a shift in the occurrence of disease classes in the transgenic lines compared to the wild type. The former all showed less plants in class 4 but more plants in classes 3 and 2, whereas the latter had most roots in class 4 (Figure 4B). We defined (partial) tolerance to clubroot as a disease index for wild type plants ≥80 and for the line to be compared as a reduction of at least about 15 units compared to the wild type. Based on these criteria, most of the *LTP* overexpressor plants were only reduced in susceptibility to clubroot, with the exception of line *LTP8-OX*, which showed a significant reduction in the disease index and a small increase in the shoot index indicating a clubroot tolerance. The transgenic antisense line *AT4G33550-AS* showed a significant higher disease index and lower shoot index compared to the wild type plants, whereas the LTP mutants with reduced LTP expression (T-DNA insertion mutants and second antisense line) did not show a remarkable difference in disease symptoms (Figure 5). No differences in disease development were visible for transgenic plants transformed with the empty vector control (EPG) compared to wild type (Figure 4).

There are several reports that the overexpression of *LTP* genes caused a reduction in disease severity. For example, *Ace-AMP1* from *Allium cepa* was overexpressed in *Oryza sativa* and conferred resistance to *Magnaporthe grisea*, *Rhizoctonia solani* and *Xanthomonas oryzae* [13], *LTP2* from *Hordeum vulgare* was overexpressed in *Nicotiana tabacum* and *A. thaliana* and conferred resistance to *Pseudomonas syringae* [52]. This led us to expect that the overexpression, and more precisely the pathogen inverse regulation, of *LTP* genes may result in reduced clubroot symptoms. It was already shown earlier that a pathogen inverse regulation of clubroot relevant genes such as cytokinin oxidases and invertase inhibitors reduce clubroot symptoms [11,53]. In contrast to clubroot resistance genes like *Crr1a* from *Brassica rapa* that encodes a TIR-NBS-LRR protein conferring most likely resistance by a gene-for-gene interaction [54], for plants with pathogen inverse *LTP* regulation, a clubroot resistance phenotype was not to be expected. Since resistance describes the prevention of infection and limitation of pathogen growth [55], it is more likely that LTPs, which belong to the class 14 of pathogenesis-related proteins that act more downstream in the recognition and signaling process following an infection, cause rather a tolerance response to the clubroot pathogen. 

Despite many reports that describe the “beneficial” function of LTPs in relation to pathogen infection there is only one recent publication that relates an enhanced susceptibility to *P. syringae* infection to *LTP3* overexpression. Contrarily, knockout of the *LTP3* gene did not result in changed susceptibility to *P. syringae* infection; however, the double knockout of *LTP3* and *LTP4* caused reduced susceptibility to *P. syringae* [56]. Since our data showed that overexpression of *LTP3* caused reduced clubroot susceptibility but overexpression of *LTP3* showed enhanced susceptibility to *P. syringae*, this indicates that the LTP function can vary between different plant–pathogen interactions.

Based on sequence similarity and *in vitro* data for some of the LTPs, the genes encoding LTP1, LTP3, LTP4, LTP8, AT5G05960, AT3G22620 and AT4G33550 are potentially involved in lipid trafficking [57]. Therefore, these LTPs may contribute to the formation of cuticular waxes in the roots, thereby influencing the natural barrier between plants and microbes in the soil. For example, an altered cuticular lipid content as the result of *LTPG1*-knockout in *A. thaliana* caused a higher susceptibility to fungal infection, as it was shown for *Alternaria brassicicola* [58].

Since the results of our infection tests using transgenic lines that overexpress the genes *LTP1*, *LTP3*, *LTP4* and *LTP8* did not show strong effects on disease symptoms, we assume that the proteins encoded by the tested *LTP* genes do not directly act against *P. brassicae* as antimicrobial compounds as it was previously described for some LTPs in the interaction with other pathogens [12,15]. Moreover, some of the LTPs we investigated could possess other beneficial features that help the infected plants deal with the pathogen. However, it cannot be ruled out that individual LTPs can substitute for each other. The analysis of gene expression of other *LTP* genes in the LTP mutants and overexpressors clearly showed that the expression of other *LTP* genes was changed in the LTP mutants compared to the wild type (Supplementary Figure S2). This complex expression pattern can maybe explain that a single *LTP* gene knockout or overexpression did not result in a clear clubroot-related phenotype. Therefore, the generation of multiple mutants should be taken into account in the future.

### 2.3. LTP Mutants Do Not Have an Altered Lipid Content

Since lipid transfer proteins were defined by their ability to transfer phospholipids between membranes *in vitro* [1], they could be involved in transferring lipids from the host plant to the pathogen to ensure its nutrition or at least influence colonization. This assumption is supported by the report that the sunflower LTP HaAP10 acted as a fatty acid shuttle between the oil body and the glyoxysome. in germinating sunflower seeds, indicating its involvement in the mobilization of lipids during germination [59]. Moreover, the lipid transfer protein AsE246 from *Astragalus sinicus* was able to bind lipids and transport plant derived lipids to the symbiosome membrane, thereby influencing nodulation of the host plant [60].

Even though the differences in disease development in some LTP overexpressors (*LTP1-OX*, *LTP3-OX*, *LTP4-OX*, *LTP8-OX*, see Figure 4) were only small, we hypothesized that this could be at least partially the result of an altered lipid composition in the galls leading to disordered lipid reserve accumulation in the pathogen. The observations that 1) all developmental stages of *P. brassicae* contain a large amount of lipids [39] and 2) one possible function of lipid transfer proteins is lipid binding and transport [1] support this hypothesis. Therefore, we have analyzed the galls of LTP mutants and overexpressors for their lipid compositions with thin layer chromatography (TLC). 

**Figure 6:**
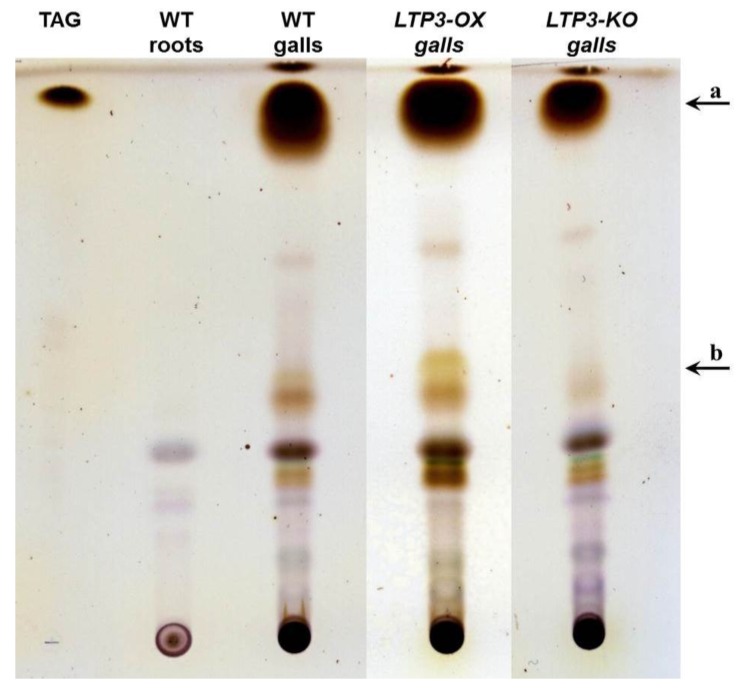
Lipid composition of control roots and galls of *A. thaliana*. Results of thin layer chromatography from non-polar lipids isolated from equal amounts of infected roots (galls) 30 days after inoculation or of healthy root material (roots) of the same age. Plant material was from wild type (WT) and LTP mutants that overexpress *LTP3* (*LTP3-OX*) and from plants with reduced *LTP3* expression (*LTP3-KO*). Two biological replicates with approximately 25 plants each were analyzed. The small differences in the fractions (**a**) and (**b**) were not consistent between biological replicates. Triacylglycerol (TAG) was used as a standard.

Lipids extracted from equal amounts of plant material were spotted on the TLC plate (Figure 6 and Supplementary Figure S3).

The results of the lipid analyses clearly showed that infected roots (galls) contained a larger amount of lipids, mainly triacylglycerols, than non-infected roots (Figure 6). Mainly the triacylglycerol fraction (fraction a in Figure 6, Supplementary Figure S3) accumulated during the infection in a time dependent manner (data not shown). This was not only the case for wild type plants, but also for all analyzed LTP mutants (Supplement Figure S3). No differences in lipid composition between the different analyzed LTP mutant lines in comparison to the wild type were observed (Figure 6 and Supplementary Figure S3). To rule this out, the analysis was done for extracts where the clubroot typical triacylglycerol concentration was in a saturation range, and as no differences were visible, we analyzed diluted extracts of all samples by TLC. In these samples, again no differences were visible between LTP mutant and wild type galls (data not shown). The small differences in the fractions a and b (indicated by arrows, Figure 6 and Supplementary Figure S3) were variable between different biological replicates. Thus, the investigated LTPs do not have an effect on the lipid content in transgenic and mutant lines. To figure out a qualitative difference in the lipid composition of the *LTP* mutants should be part of further investigation using for example MS-MS analyses.

Many microscopy-based data describe large lipid droplets as storage compounds in all developmental stages of *P. brassicae*. So far, the composition of these storage lipids is not well known. Our results show that triacylglycerols accumulate in the galls of infected plants. In addition, Sundelin *et al*. [61] showed that arachidonic acid is the most abundant fatty acid in resting spores and is only found in root galls of infected plants. Data from the *P. brassicae* genome sequence suggest that the pathogen is not able to synthesize fatty acids *de novo*, but uses the microsomal elongase pathway to modify plant derived fatty acids [62]. Therefore, the mobilization of fatty acids from the host plant is necessary and plant lipid transfer proteins could be involved in carrying fatty acids between the host plant and *P. brassicae*. However, the LTPs tested in this study did not influence the amount of triacylglycerols and are therefore perhaps not responsible for the translocation of fatty acids.

### 2.4. Lipid Transfer Proteins Influence the Sensitivity to Salt Stress

Roots of plants are subject to many biotic, but also abiotic influences that can interact with each other. For example, some features in older root galls after *P. brassicae* infection resemble drought stress conditions [37], which in turn affect plant (root) growth via nutrient and water uptake. During the late phase of clubroot infection, the colonized roots show disrupted vascular bundles and as a result the upper plant parts display typical drought stress symptoms, such as wilted leaves [37]. From the transcripts that were more than 20-fold upregulated between infected young roots and older roots in a microarray [11], *ca.* 20% were associated with ABA or drought stress response [36]. ABA is also involved in the regulation of the response to other abiotic stress factors [23,25]. Among these abiotic factors influencing root growth are salt and osmotic stressors [63,64]. The response of plants to drought and salt stress is in many aspects very similar since salinity reduces the capacity of the plant for water uptake [24].

**Figure 7:**
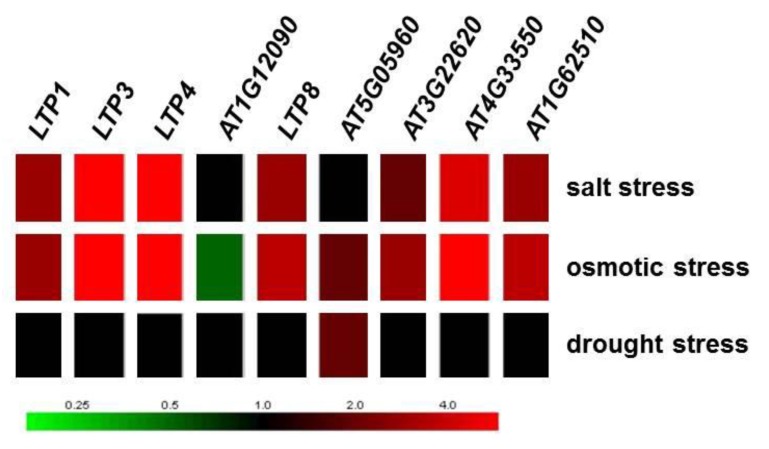
Regulation of *LTP* gene expression due to salt, osmotic and drought stress based on microarray data (data taken from Genevestigator [49]).

It was shown that many *LTP* genes from different species are induced upon salt, osmotic and drought stress treatment [26,65]. From *A. thaliana,* the genes *LTP1*, *LTP3*, *LTP4 LTP8*, *AT3G22620*, *AT4G33550* and *AT1G62510* were induced upon salt and osmotic stress treatment but not drought (Figure 7; data taken from Genevestigator [49]), which allowed the assumption that an altered *LTP* gene expression should result in changes in sensitivities to salt and osmotic stress. A subset of *LTP* genes (*LTP3*, *LTP4*, *AT1G62510*, *AT3G22620*, *AT3G33550*) was also induced by ABA (Figure 2). Especially, *LTP3* was shown to be induced by ABA and NaCl [56]. Therefore, we have analyzed plant root growth under drought, salt and osmotic stress conditions to elucidate a possible role for LTPs in the mutants and transgenic lines. Drought stress did not result in significant phenotypical changes in any of the mutant or overexpressor lines generated for the different *LTP* genes compared to wild type (data not shown).

**Figure 8:**
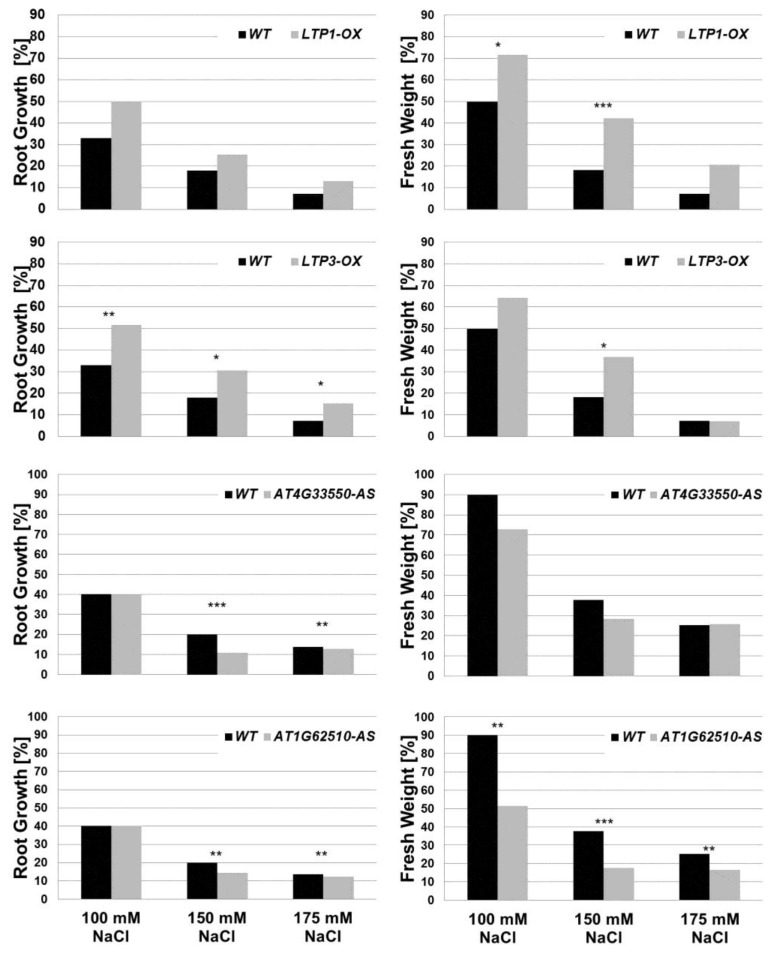
Growth reduction due to salt stress conditions. Root growth and whole plant fresh weight from wild type (WT) and LTP overexpressor and mutant lines (*LTP1-OX*, *LTP3-OX*, *AT4G33550-AS* and *AT1G62510-AS*) in response to salt stress are shown. To calculate the root growth and fresh weight (in %), the values from unstressed plants (0 mM NaCl) were set to 100%. Therefore, the graphs show the growth reduction due to salt stress. Since these graphs show a ratio based on the mean value, the standard deviation could not be plotted on this graph. n ≥ 50. Asterisks indicate a significant difference (for * *p* < 0.05; ** *p* < 0.01; *** *p* < 0.001). bar = 1 cm.

It is well known that *A. thaliana* is a salt sensitive plant, which shows a strong growth reduction due to salt stress [24,63]. To simulate salt stress conditions, plants were cultivated on Murashige and Skoog (MS) agar plates containing different concentrations of NaCl (see Section 3.1). Root length and fresh weight of plants were determined 14 and 21 days after sowing (Figure 8, Supplementary Figure S4). As expected, plants grown on media containing 100, 150 and 175 mM NaCl exhibited shorter roots and lower fresh weight compared to unstressed plants on salt free medium. The LTP mutants overexpressing *LTP1* (*LTP1-OX*) or *LTP3* (*LTP3-OX*) grew better than wild type plants under salt stress conditions, which was shown by the development of a statistically significant larger root system and production of more fresh weight compared to wild type plants (Figure 8). In comparison, the other *LTP* overexpression lines (*LTP4-OX*, *AT1G12090-OX*, *LTP8-OX*) did not show statistically significant differences in growth compared to wild type plants (Supplement Figure S4). The antisense lines (*AT4G33550-AS* and *AT1G62510-AS*) showed a stronger growth reduction on NaCl containing media compared to wild type plants (Figure 8). All LTP knockout plants tested did not show a distinct growth effect compared to the wild type (Supplement Figure S5). These results suggest that *LTP1* and *LTP3* are involved in the plant's response during salt stress. This is supported by the recent finding that plants overexpressing *LTP3* have an increased endogenous ABA level. Moreover, it was concluded that *LTP3* is a positive regulator of the ABA pathway and is involved in biotic and abiotic stress responses [56].

To simulate osmotic stress, all LTP mutant and overexpressor lines as well as wild type plants were cultivated on MS medium containing different concentrations of mannitol (300 mM, 350 mM). However, the results did not give a clear indication that any of the LTPs investigated here influenced the plant growth during these osmotic stress conditions (see Supplementary Figures S6 and S7 for details).

Only a few publications are available that describe an altered sensitivity to salt and osmotic stress as a result of modulated *LTP* gene expresion. For example, pepper *CALTPI* was induced upon salt stress and overexpression in *A. thaliana* resulted in better growth during salt stress [10,66]. It was speculated that this LTP could be involved in the cellular reaction to stress induced damages or in the formation of a protective layer towards dessication [66]. *TdLTP4* from durum wheat was also induced under salt stress conditions and overexpression in *A. thaliana* led to promoted growth under this stress factor [67]. AZI1 from *A. thaliana* is a LTP related hybrid proline rich protein (HyPRP) that improve salt stress tolerance [68]. It was shown that AZI1 is a target of MPK3 (mitogen-activated protein kinase 3) *in vitro*. Despite these hints from the literature that the LTPs are somehow involved in the adaptation to various stressors, their detailed function *in vivo* remains dubious. The LTP mutants investigated so far showed some changes in their sensitivity to salt stress, but an explanation of how this effect is mediated by LTPs is still missing.

## 3. Experimental Section

### 3.1. Plant Material and Growth Conditions

*A. thaliana* wild type Columbia (Col-0) was used in all experiments. The *LTP* transgenic lines generated and the T-DNA insertion lines (obtained from the Nottingham Arabidopsis Stock Centre) are also based on the *A. thaliana* wild type Col-0 background. Plant cultivation was always carried out under long day conditions (16 h light, 23 °C/8 h dark, 18 °C) in the greenhouse. For the infection with *P. brassicae*, plants were cultivated in a soil-sand mixture (3:1; *w*:*w*) in planting pots (20 cm × 30 cm, 30 plants in each pot).

For salt and osmotic stress treatments, plants were cultivated in petri dishes (Φ 92 mm, 7 plants each) on MS-medium containing different concentrations of NaCl (100, 150 and 175 mM) or mannitol (300 and 350 mM).

### 3.2. Infection with Plasmodiophora brassicae and Disease Rating

The *P. brassicae* isolate e_3_ [43] was used in all experiments. Propagation of the pathogen was carried out in *B. rapa* plants. Isolation of spores of the pathogen was done as described earlier [69].

For the infection with *P. brassicae*, plants were cultivated as described in Section 3.1. Fourteen-day-old *A. thaliana* seedlings were inoculated by pipetting 2 mL of a spore suspension (10^5^ spores/ 1 mL in 50 mM KH_2_PO_4_, pH 5.5) on the soil around each seedling.

Disease rating was carried out 26–28 days after inoculation by taking out the roots from the soil and the disease symptoms were evaluated according to Siemens *et al*. [70]. Based on the severity of the disease symptoms, the plants were categorized into five different disease classes, which were characterized as follows (see also Figure 3). Plants in class 0 did not show any disease symptoms. If only minor swellings at the minor and/or secondary roots appeared and the typical root structure was still present, plants belonged to class 1. When the primary root was visibly thickened, the fine roots and lateral roots were reduced and also thickened, plants were grouped into class 2. Plants that belonged to class 3 were characterized by a greatly reduced root system, where galls were clearly visible at primary and secondary roots; fine roots were no longer visible, and in part the hypocotyl also showed gall development. If the roots consisted of one large brownish gall, these plants belonged to class 4. Based on this classification, a Disease Index (DI) was calculated according to the following formula:

$$\text{Disease Index (DI)}=\frac{\left(1n_{1}+2n_{2}+3n_{3}+4n_{4}\right)\cdot 100}{\text{4Nt}}$$

n_1_ to n_4_ = number of plants in the different disease classes

Nt = Number of all tested plants

Additionally, the shoot index (ratio between fresh weight of infected plants/not infected plants was calculated.

### 3.3. RNA Extraction and Semi-Quantitative RT-PCR

Total RNA from plant material (~200 mg) was isolated using Trizol^®^Reagent (Life Technologies; Thermo Fisher Scientific Inc., Waltham, MA, USA), according to the manufacturer’s instructions.

To eliminate DNA contaminations, total RNA was treated with DNaseI (DNA-free™ DNase Treatment and Removal Reagents, Life Technologies; Thermo Fisher Scientific Inc.) according to the manufacturer’s instructions. For cDNA synthesis 3–5 μg total RNA and the M-MLV-Reverse Transcriptase (Life Technologies; Thermo Fisher Scientific Inc.) was used according to the manufacturer’s instructions.

For the semi-quantitative expression analyses, the *A. thaliana* elongation factor 1B gamma (*AT1G09640*) or the Histone H2A (*AT1G52740*) were used as reference genes. The corresponding primers span one intron to guarantee that the amplificates are only based on cDNA as templates. Each PCR program consisted of 5 min denaturation at 95 °C, 20–35 cycles with 1 min at 95 °C, 1 min annealing at primer specific temperature (see Supplementary Table S1) and extension at 72 °C with 1 min per 1 kb product length and a final elongation step at 72 °C for 10 min. For the PCR amplification, 20 μL reactions were used containing reaction buffer, 0.2 μM dNTP’s (Fermentas; Thermo Fisher Scientific Inc.), specific amounts of magnesium (see Supplementary Table S1), 0.5 μM for each oligonucleotide and one unit Taq-Polymerase (Life Technologies; Thermo Fisher Scientific Inc.).

The program Image J was used to calculate the regulation of transcript levels by determining the intensity of the respective PCR product. Normalization of *LTP* transcripts was done with the reference genes mentioned above within the same experiment. Fold-changes in *LTP* mutants and transgenic lines were calculated based on expression values in the wild type.

### 3.4. Generation of Transgenic Arabidopsis thaliana Plants

To generate transgenic *A. thaliana* plants with changed LTP gene expression, the root specific promoter from the *PYK10* gene [51] was cloned in front of the *LTP* gene in sense (overexpression) or antisense (silencing) orientation, followed by the nos terminator. Constructs for plant transformation were made in the pGreen 0229 vector [71], whereas the *LTP* genes alone were first cloned in the pGEM^®^-T Easy vector (Promega GmbH, Mannheim, Germany). Correctness of the constructs was verified by sequencing.

The pGreen vector with the final construct was co-transformed with pSoup [71], in the *Agrobacterium tumefaciens* strain EHA105 [72]. Agrobacteria harboring these two plasmids were used to transform *A. thaliana* wild type plants using the floral dip method [73]. After transformation, seeds were collected and germinated on soil. Positive transformants were selected using the herbicide BASTA (0.52 mg/L) as marker. Seeds from these transgenic plants were collected and used for further experiments. Since these plants were not exclusively homozygote, the use of BASTA for elimination of wild type plants in the F2 and F3 generation was necessary. Therefore plants growing on soil were sprayed with BASTA until all wild type seedlings were eliminated. If the plants were germinated on MS agar plates, 100 μM Phosphinothricin was included. To rule out an effect of BASTA/Phosphinothricin plants transformed with an empty pGreen vector (EPG) were used as a control.

The selected transgenic lines were tested with PCR to confirm the integration of the constructs. Semi-quantitative expression analyses for the inserted transgene were also carried out.

### 3.5. Verification of T-DNA Insertion Mutants

Seeds for LTP T-DNA insertion lines were obtained from the Nottingham Arabidopsis Stock Centre (NASC). Homozygote seeds were selected using PCR with primers flanking the insertion site (see Supplementary Table S2). Additionally, expression of the disrupted gene was also tested with semi-quantitative expression analyses.

### 3.6. Lipid Extraction and TLC Analytic

The extraction of lipids from plant material was done according to Matyash *et al*. [74]. For each isolation, 50–400 mg of gall material and 100–200 mg of control roots were used.

Plant material was ground in a mortar with a pestle, and 15 μL methanol per 1 mg fresh weight was added. After homogenization, the 3.3-fold volume tertiary butyl methyl ether (MTBE) was added and the mixture was incubated for 1 h at 10 °C under continuous shaking. After the 0.2-fold volume water was added, the mixture was again incubated for 15 min at 10 °C. After centrifugation (1 min, 5000× *g*), the upper phase was removed and the lower phase was extracted with 0.2-fold MTBE/methanol/water (10:3:2.5; *v*:*v*:*v*) for 15 min at 10 °C. After centrifugation (1 min, 5000× *g*), the upper phase was collected and dried under a stream of nitrogen. The dried lipids were resolved in a defined amount of MTBE/methanol (10:3; *v*:*v*) to make the different samples comparable. For seeds, 100 μL/10 mg, and for gall material and roots, 1 μL/1 mg fresh weight was used.

The lipid composition was analyzed using thin layer chromatography. Equal amounts of extracts (based on the fresh weight used for extraction) were spotted on silica gel 60_F254_ plates and developed in hexane:ethylacetate:acetic acid (80:20:1; *v*:*v*:*v*). Lipids were visualized by spraying with 20% sulfuric acid followed by 20 min incubation at 120 °C.

### 3.7. Statistical Analyses

The program PASW Statistics 18 (IBM) was used for all statistical analyses. Since all plants cultivated in one petri dish are interdependent, the Intra-Class-Correlation was included in the tests. All datasets were checked for normal distribution using the Kolmogorov-Smirnov-Test. Finally, a parametric test was used (mixed linear model, without interplay effects). To analyze the data from the phytopathological studies, the H-Test according to Kruskal and Wallis was used.

### 3.8. Re-Analysis of Available Microarray Experiments

The AGI numbers of transcripts for selected genes were from the TAIR database [6]. Transcript levels were compared for different abiotic stressors and hormone treatment by using the Genevestigator database [49] and for control and *P. brassicae* infected roots using the microarray experiment E-MEXP-254 [11].

## 4. Conclusions

**Figure 9:**
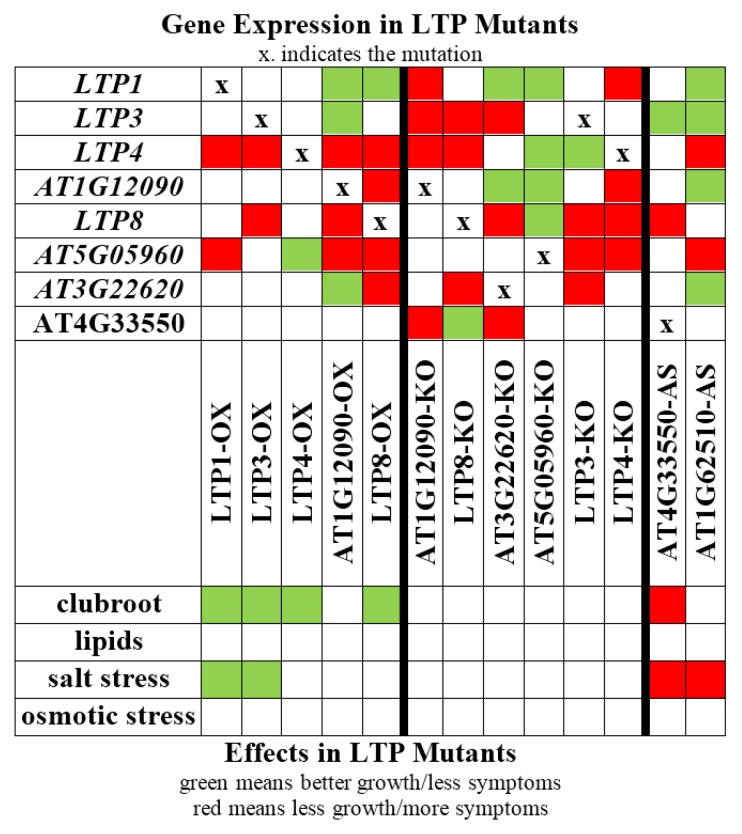
Overview of effects caused by a single LTP mutation or overexpression of one *LTP* gene. All effects found for the different analyzed LTP mutants are shown. OX: overexpression, KO: knockout or knockdown, AS: antisense.

The aim of our study was to shed light on the function of the still enigmatic LTP family. We have analyzed plants that overexpress one LTP gene and plants with reduced LTP gene expression with regard to their phenotype during clubroot development, including lipid composition in galls, salt and osmotic stress conditions (Figure 9).

While the LTP mutants did not reveal altered root and plant growth, we could show that some LTPs are involved in clubroot development. Overexpression of *LTP1*, *LTP3*, *LTP4* and *LTP8* led to reduced clubroot susceptibility, and the reduced expression of *AT4G33550* can cause higher susceptibility. Moreover, it was shown that plants that overexpress *LTP* genes (*LTP1*, *LTP3*) grew better on NaCl containing media indicating a role in the adaptation to this abiotic stress factor. Additionally, we also observed a stronger growth reduction due to NaCl treatment in two lines with reduced *LTP* expression (*AT4G33550*, *AT1G62510)*. Older clubbed roots show symptoms similar to drought stress, and drought and salt stress signaling pathways share similar components. Two LTPs (LTP1 and LTP3) might be involved in the cross-talk of the different pathways, since their overexpression resulted in more tolerant plants to both the clubroot pathogen and salt stress (Figure 9), even though the experimental conditions between the two datasets certainly are different.

The results presented here can only refer to physiological processes in which the tested LTPs are involved. The details of their behavior remain a secret. Since LTPs form a large protein family, a lot of coordinated experiments will be necessary to connect one LTP with a specific function.

## Supplementary Materials

Supplementary materials can be accessed at: http://www.mdpi.com/2223-7747/5/1/2/s1.
