# Supplementary material for: Response of Arabidopsis thaliana Roots with Altered Lipid Transfer Protein (LTP) Gene Expression to the Clubroot Disease and Salt Stress
Source: Plants (Basel). 2015 Dec 24;5(1):2. doi: 10.3390/plants5010002 (PMC4844412; doi:10.3390/plants5010002)
Supplement: Supplementary file 1 [file plants-05-00002-s001.pdf]

Supplementary Materials

Table S1. Oligonucleotide sequences and PCR conditions used to amplify the indicated genes. TA = annealing temperature; gDNA = genomic DNA; cDNA = complementary DNA; c = concentration.

Gene	Oligo Sequence (5'-->3')	T_A in ℃	c [MgCl] in mM	PCR-Product Size
EF	tcccttcaacactcctttatagc<br>aacagtctatgcgacacgtca	58	2.5	gDNA: 2354 bp<br>cDNA: 1500 bp
H2A	cggggaaaggtgctaaaggtt<br>aaatgcctcggcgagatacgt	55	2.5	gDNA: 690 bp<br>cDNA: 282 bp
LTP1	ctccaaggtggtgtcattcc<br>cccatataacaagaacaccacaa	57	2.5	gDNA: 450 bp<br>cDNA: 380 bp
LTP3	tgttatcacccaaaaagaagttca<br>ttattattacgttcgtatgcgttgg	57	2.5	gDNA: 703 bp<br>cDNA: 610 bp
LTP4	tatcacccaaaagagaagagca<br>acacaagtatacaacataacaaagc	54	2.5	gDNA: 649 bp<br>cDNA: 510 bp
AT1G12090	cccaattcactcacaacctagc<br>atcaccccaatgaacaccag	58	1.5	gDNA: 630 bp<br>cDNA: 630 bp
LTP8	gcaacaacaaagaaacccсta<br>ttaggacaagatggaccattga	58	2.5	gDNA: 885 bp<br>cDNA: 448 bp
AT5G05960	tcatcactcaagaatggaaacc<br>acgtctattgctttctgtctgc	56	2.5	gDNA: 390 bp<br>cDNA: 479 bp
AT4G22610	taaatccaagccctcacctc<br>cagcaacaactacgatcatgc	55	2.5	gDNA: 501 bp<br>cDNA: 501 bp
AT3G22620	cacacttcaaacacaaaaccac<br>cttattccacaaagcaatgacc	67	2.5	gDNA: 979 bp<br>cDNA: 790 bp
AT4G33550	cggaccaaatattcgcattc<br>ccggaatggtgtaacctataaca	58	2.5	gDNA: 490 bp<br>cDNA: 490 bp
AT1G62510	tcccaattcacacataccaaag<br>ggtagaaatcatcttgtctgtcca	27	1.5	gDNA: 605 bp<br>cDNA: 605 bp
Pb Actin	agctggcgtacgtggcgcag<br>ccttgacgcgcatcgacgac	64	2.5	gDNA: 342 bp<br>cDNA: 342 bp
nos-terminator	tatagcggccgcggatcgttcaaacatttggcaata<br>gcgcgagctcatctagtaacataga	55	2.5	273 bp

Table S2. Oligonucleotide sequences and PCR conditions used to verify the T-DNA integration. TA = annealing temperature; c = concentration.

T-DNA Insertion Line Acc. No/ Disrupted Gene	Oligos Used 5'-->3'	Site of T-DNA Insertion	T_A in °C	c [MgCl] in mM	PCR-Product Size without T-DNA
N647582/ *AT1G12090*	cgatcctgtttcgcgtagat atcaccccaatgaacaccag	Promoter	60	2,5	961
N520925/ *LTP8*	tgttgaaagagcttgtagtaatgg gcaacaacaaagaaacccccta	3' utr	58	2,5	1535
N648038/ *AT3G22620*	tccgaatacgacctacaatga cttattccacaaagcaatgacc	Promoter/ 5' utr	58	2,5	1564
N527420/ *AT5G06960*	ttgaggactggagaaaagga acgtctattgctttctgtctgc	Promoter	58	2,5	872
N500561/ *LTP4*	aaggattcttgcagtttcat acacaagtatacaacataacaaagc	Promoter	58	2,5	2093
N595248/ *LTP3*	tgttatcacccaaaaagaagttca ttattattacgttcgtatgcgttgg	Exon	58	2,5	703

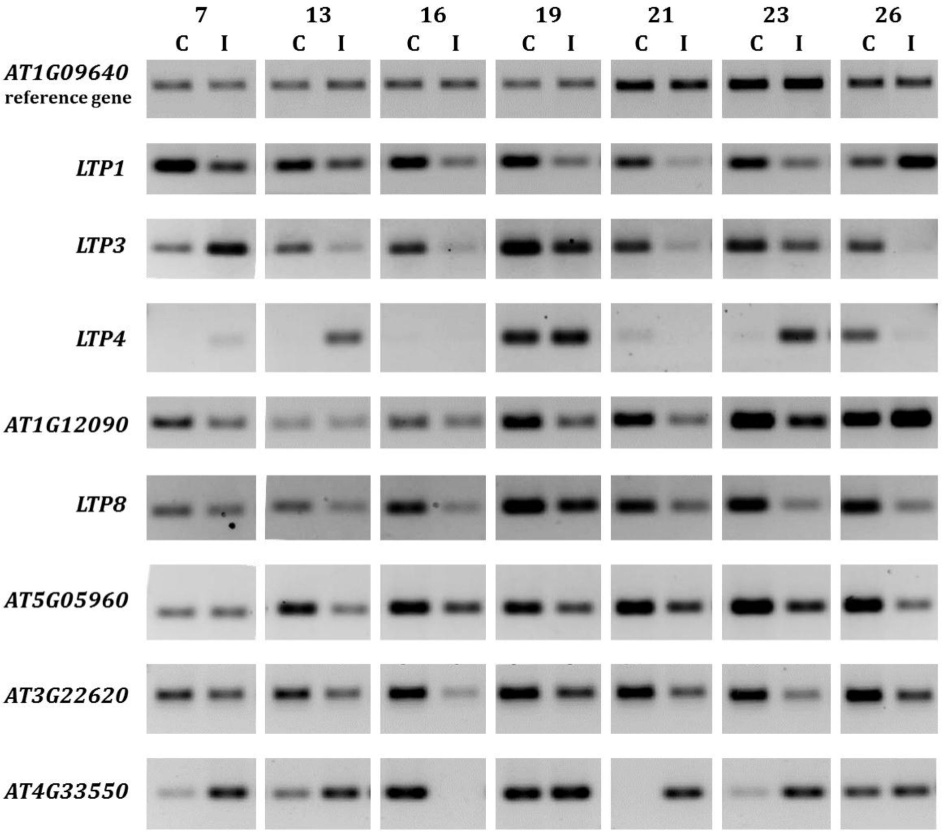

Figure S1. Expression of selected *LTP* genes in clubroot infected *A. thaliana* roots. The expression for some *LTP* genes during clubroot infection between 7 and 26 days after inoculation is shown. For the gene *AT1G62510* the expression was not detectable. For infection a field isolate [44] was used. C = control; I = inoculated.

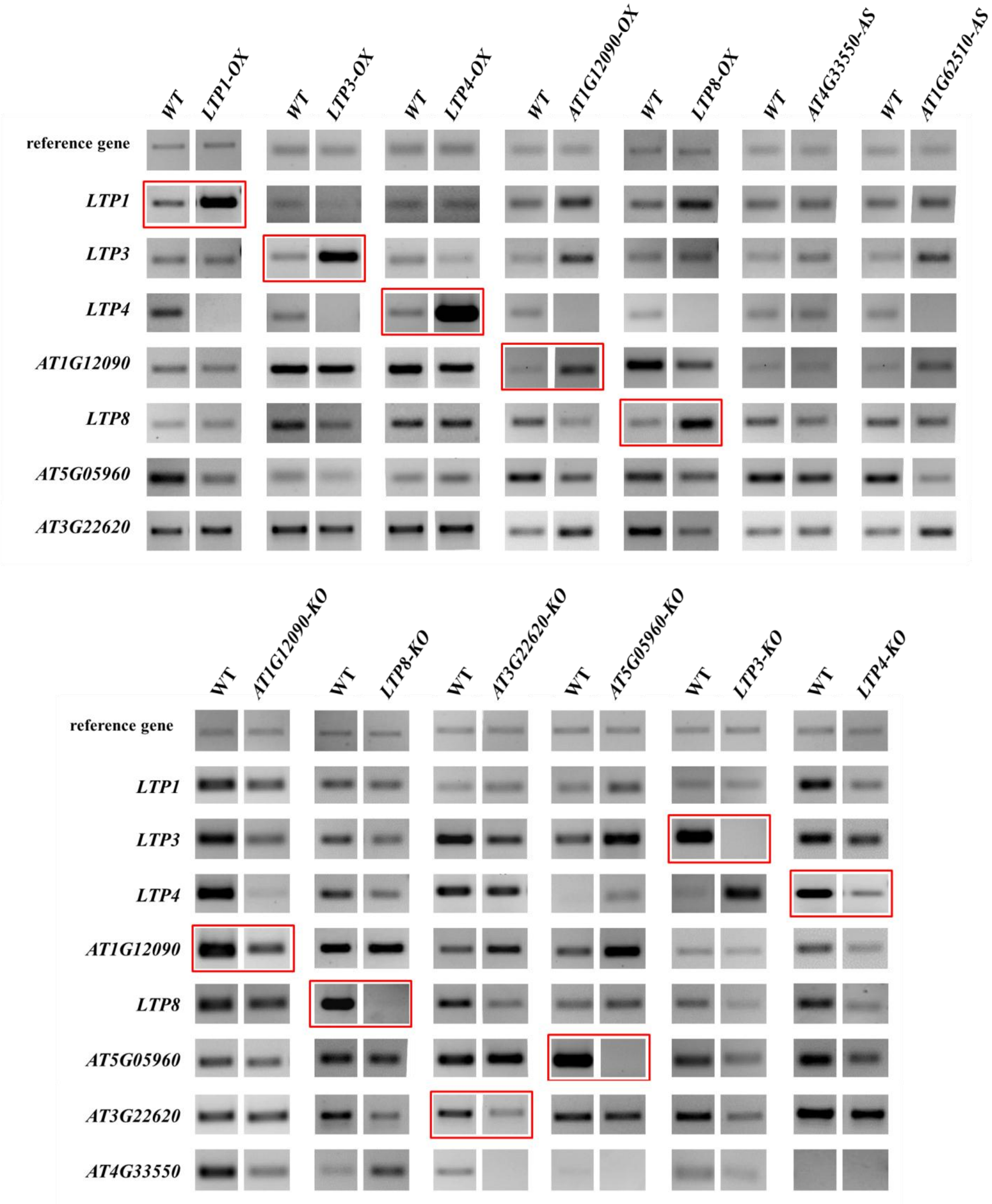

Figure S2. Expression of some *LTP* genes in roots of LTP mutants. The expression for the indicated *LTP* genes (left) in comparison to the wild type (WT) is shown. The elongation factor 1B gamma (*AT1G09640*) and histon H2A (*AT1G52740*) were used as reference genes. For the semi-quantitative expression analyses the RNA was extracted from 24-day-old *A. thaliana* roots. The red squares mark the expression of the overexpressed (OX) or down-regulated (KO) gene. For *AT1G62510* and *AT4G33550* the expression in the transgenic plants as well as in the wild type plants and for *AT1G62510* in the T-DNA insertion lines and their wild type control the gene expression was not detectable.

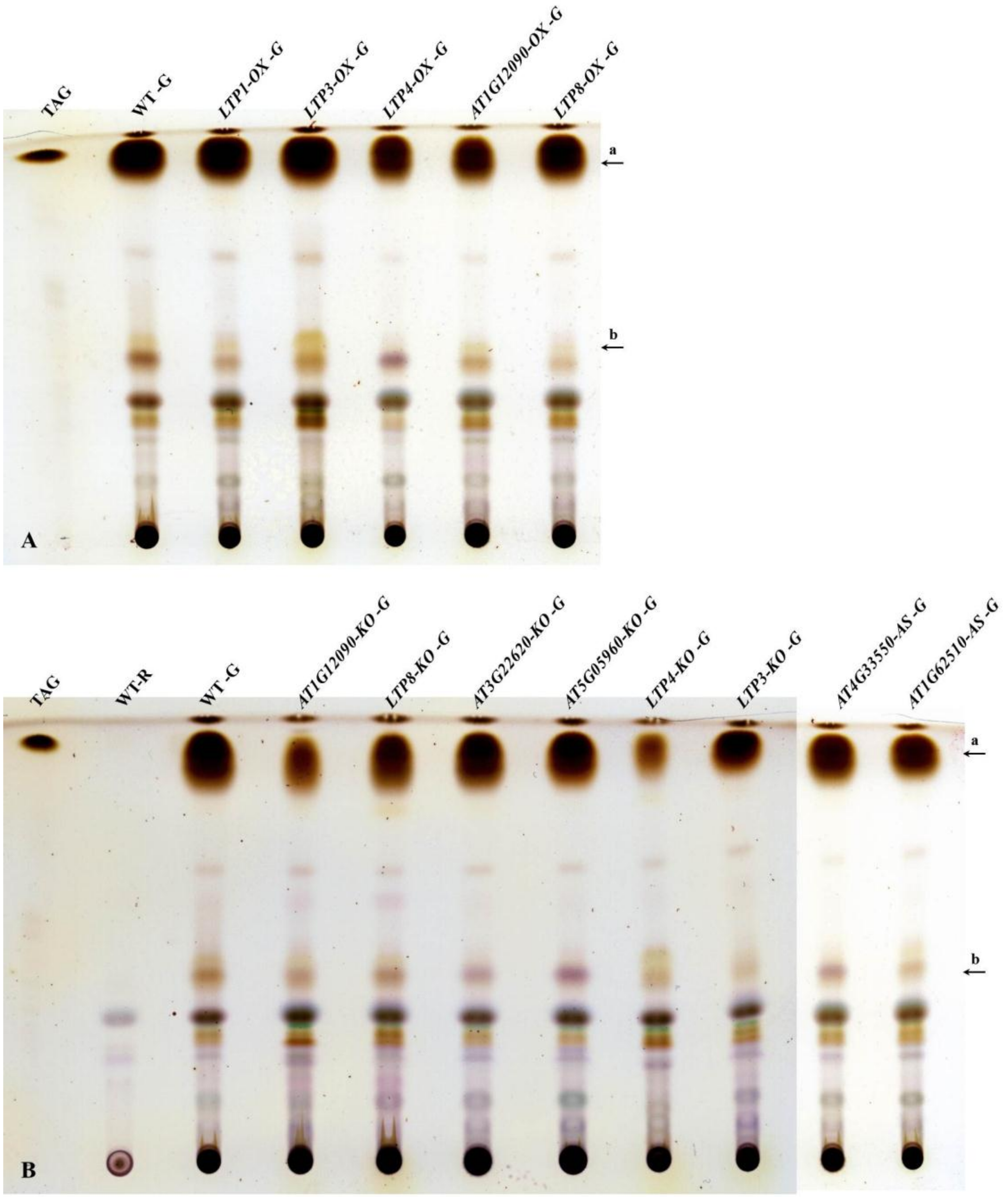

Figure S3. Lipid composition of uninfected roots and galls of *A. thaliana*. Results of thin layer chromatography from non-polar lipids isolated from equal amounts of infected roots (galls) 30 days after inoculation or of healthy root material of the same age. Plant material was from wild type (WT) and LTP mutants that (**A**) overexpress (OX) the genes *LTP1*, *LTP3*, *LTP4*, *AT1G12090* and *LTP8* and with reduced LTP gene expression (**B**) from T-DNA-insertion lines (KO) for the genes *AT1G12090*, *LTP8*, *AT3G22620*, *AT5G05960*, *LTP4*, *LTP3* and antisense lines (AS) for the genes *AT4G33550* and *AT1G62510*. Two biological replicates with approx. 25 plants each were analyzed. Triacylglycerol (TAG) was used as a standard. R = uninfected root; G = gall (infected root). For explanation of bands a and b see main text.

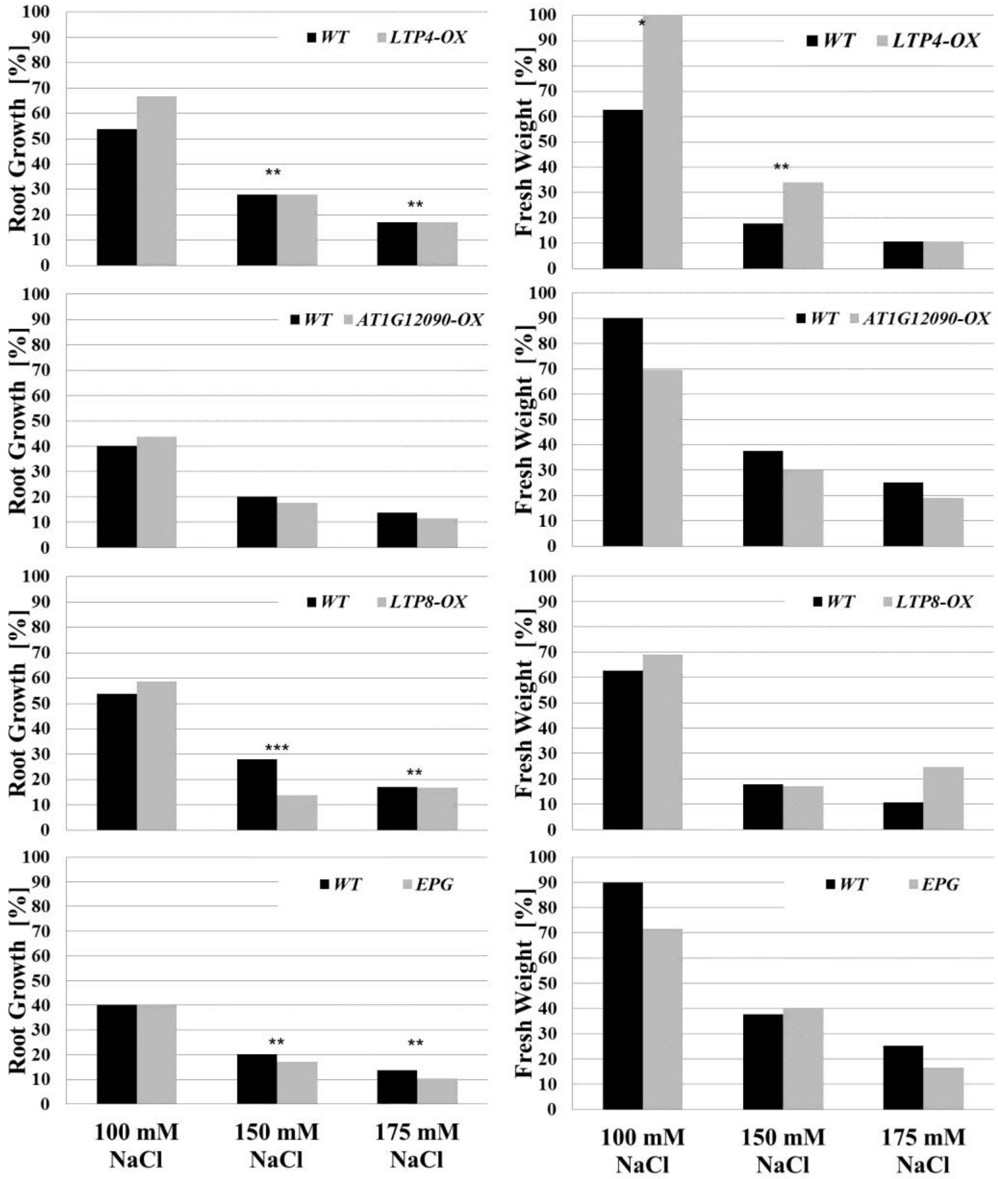

Figure S4. Growth reduction due to salt stress conditions. Root growth and whole plant fresh weight from wild type (WT), LTP mutants (*LTP4-OX*, *AT1G12090-OX*, *LTP8-OX*) and the empty vector control (*EPG*) in response to salt stress are shown. To calculate the root growth and fresh weight (in %) the root growth and fresh weight from unstressed plants (0 mM NaCl) were set to 100%. Therefore the graphs show the growth reduction due to salt stress. $n \geq 50$. Asterisks indicates a significant difference (for ** $p < 0.01$; *** $p < 0.001$). OX: overexpression of the indicated gene.

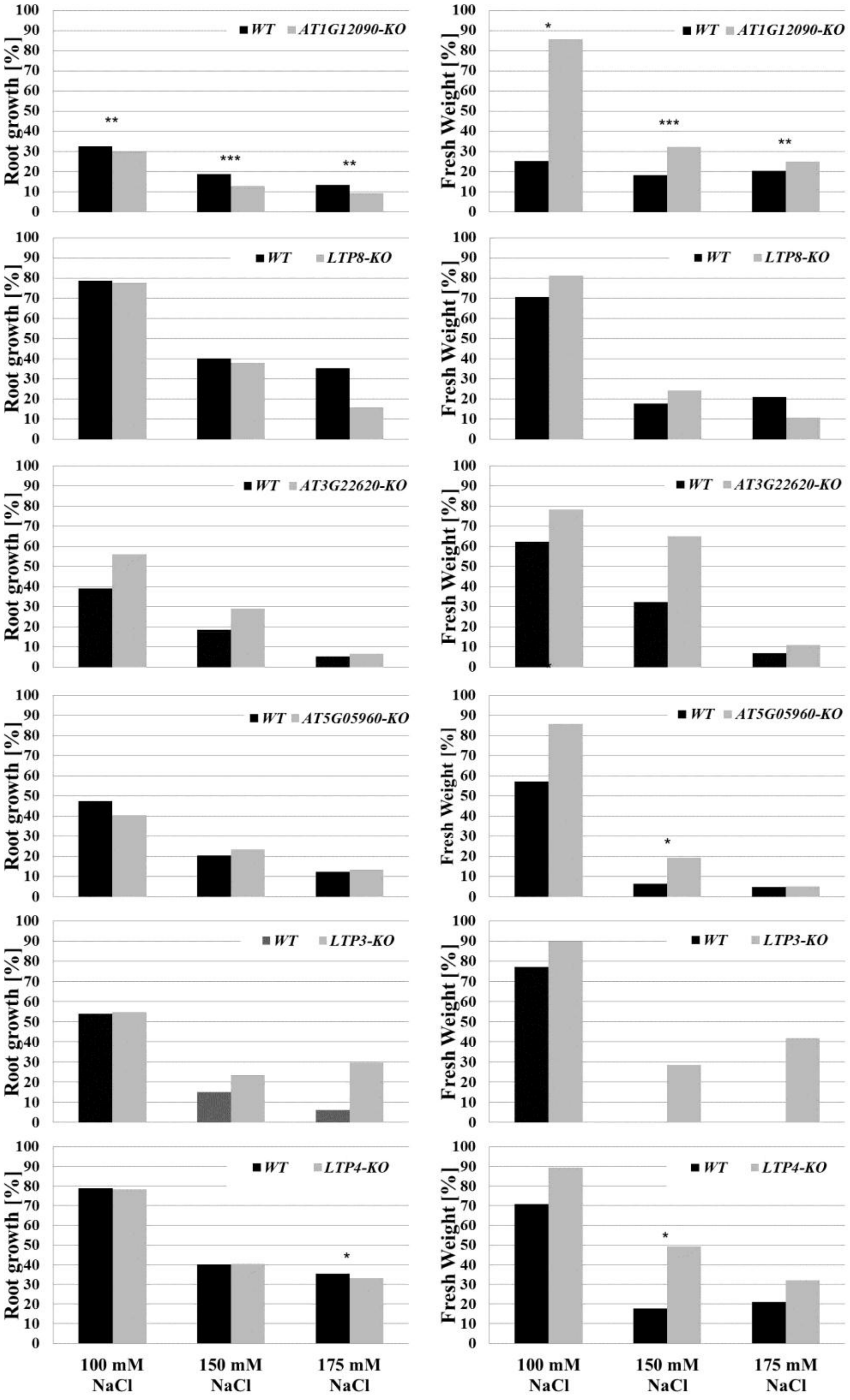

Figure S5. Growth reduction due to salt stress conditions. Root growth and whole plant fresh weight from wild type (WT) and LTP mutants (*AT1G12090-KO*, *LTP8-KO*, *AT3G22620*, *AT5G05960-KO*, *LTP3-KO*, *LTP4-KO*) in response to salt stress are shown. To calculate the root growth and fresh weight (in %) the root growth and fresh weight from unstressed plants (0 mM NaCl) were set to 100%. Therefore the graphs show the growth reduction due to salt stress. Since these graphs show a ratio based on the mean value the standard deviation could not be plotted on this graph. $n \geq 30$, KO: knockout or knockdown of the indicated gene. Asterisks indicates a significant difference (for * $p < 0.05$; ** $p < 0.01$; *** $p < 0.001$).

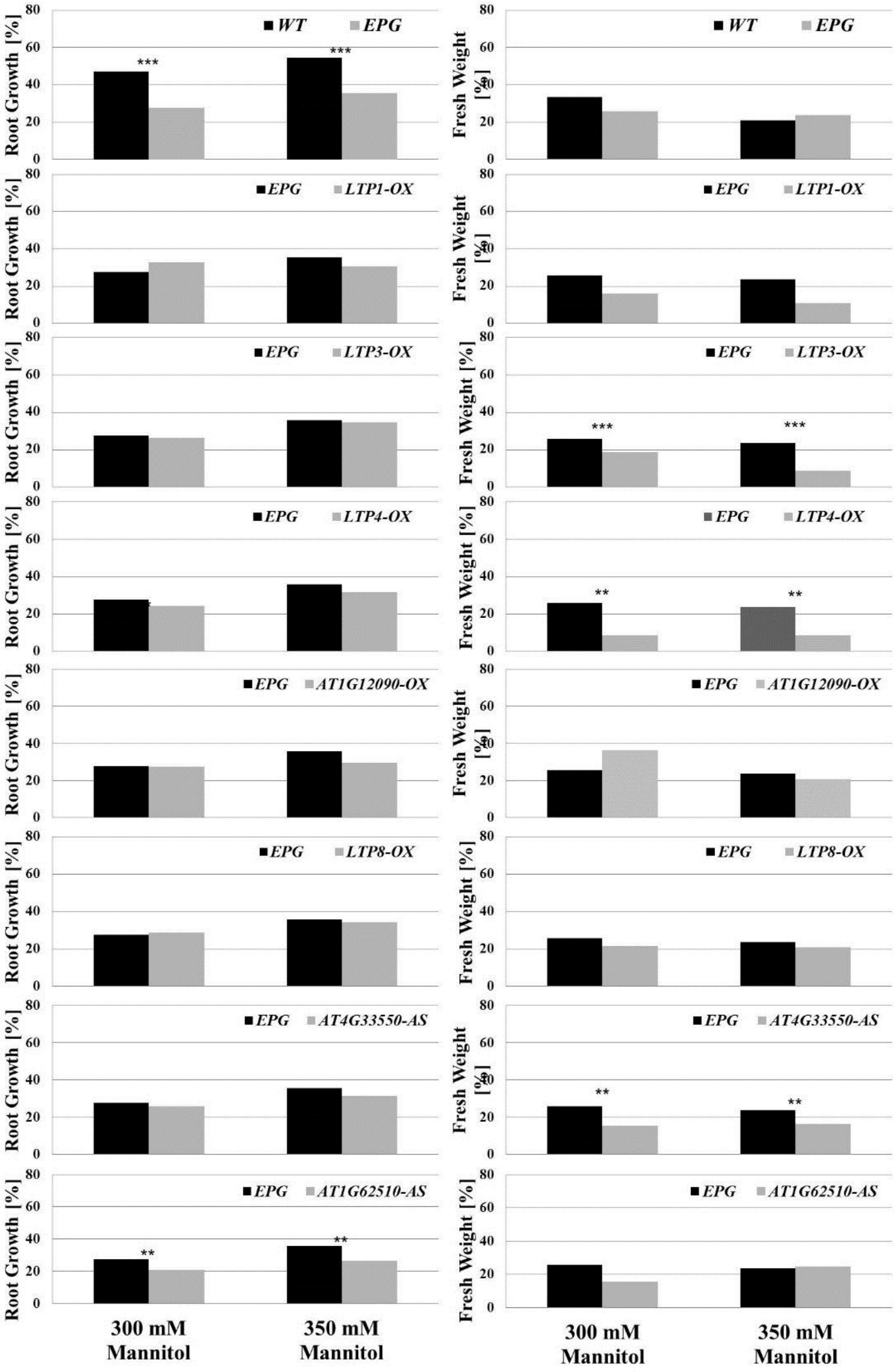

Figure S6. Growth reduction due to osmotic stress conditions. Root growth and whole plant fresh weight from wild type (WT) and LTP mutants (*LTP1-OX*, *LTP3-OX*, *LTP4-OX*, *AT1G12090-OX*, *LTP8-OX* and *AT4G33550-AS*, *AT1G62510-AS*) in response to mannitol treatment (300 mM, 350 mM) are shown. To calculate the root growth and fresh weight (in %) the root growth and fresh weight from unstressed plants (0 mM NaCl) were set to 100%. Because of the obvious differences between the vector control (EPG) and the wild type plants, the mutants were compared to the vector control plants (EPG). Since these graphs show a ratio based on the mean value the standard deviation could not be plottet on this graph. n ≥ 30, OX: overexpression of the indicated gene; AS: silencing of the indicated gene using antisense technique. Asterisks indicates a significant difference (for ** $p < 0.01$; *** $p < 0.001$).

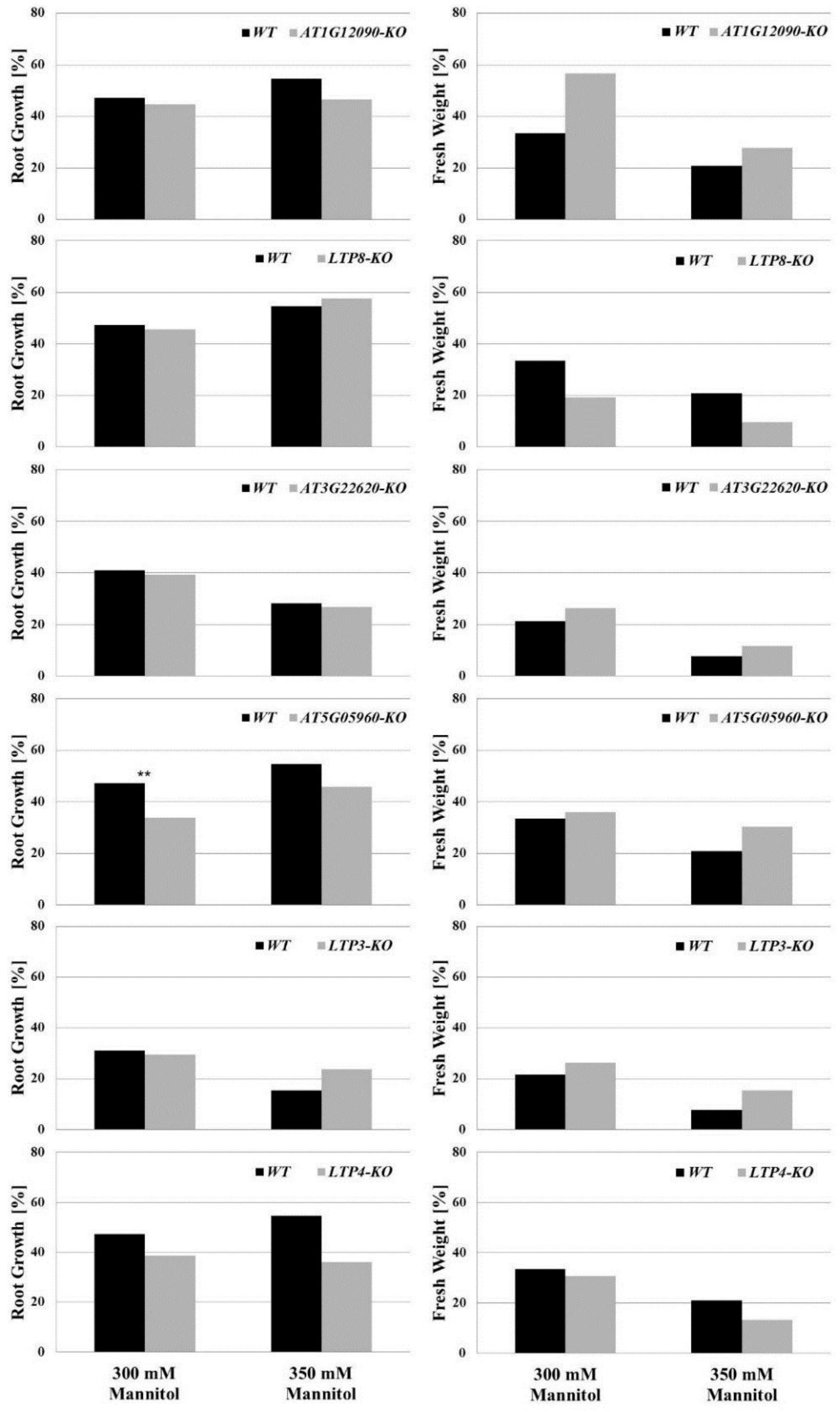

Figure S7. Growth reduction due to osmotic stress conditions. Root growth and whole plant fresh weight from wild type (WT) and LTP mutants *(AT4G33550-AS*, *AT1G62510-AS*, *AT1G12090-KO*, *LTP8-KO*, *AT3G22620-KO*, *AT5G05960-KO*, *LTP3-KO*, *LTP4-KO*) in response to mannitol treatment (300 mM, 350 mM) are shown. To calculate the root growth and fresh weight (in %) the root growth and fresh weight from unstressed plants (0 mM NaCl) were set to 100%. Since these graphs show a ratio based on the mean value the standard deviation could not be plotted on this graph. Because of the obvious differences between the vector control (EPG) and the wildtype plants (see S7). n ≥ 30, KO: knockout or knockdown of the indicated gene, Asterisks indicates a significant difference (for ** $p < 0.01$).
